# Manipulation of the inflammatory reflex as a therapeutic strategy

**DOI:** 10.1016/j.xcrm.2022.100696

**Published:** 2022-07-19

**Authors:** Mark J. Kelly, Caitríona Breathnach, Kevin J. Tracey, Seamas C. Donnelly

**Affiliations:** 1 Department of Clinical Medicine, Trinity College Dublin, Dublin, Ireland; 2 Tallaght University Hospital, Dublin, Ireland; 3 Center for Biomedical Science and Bioelectronic Medicine, Feinstein Institutes for Medical Research, Northwell Health, 350 Community Drive, Manhasset, NY 11030, USA

**Keywords:** inflammatory reflex, cholinergic anti-inflammatory pathway, inflammatory disease, α7 nicotinic acetylcholine receptor, vagus nerve stimulation

## Abstract

The cholinergic anti-inflammatory pathway is the efferent arm of the inflammatory reflex, a neural circuit through which the CNS can modulate peripheral immune responses. Signals communicated via the vagus and splenic nerves use acetylcholine, produced by Choline acetyltransferase (ChAT)+ T cells, to downregulate the inflammatory actions of macrophages expressing α7 nicotinic receptors. Pre-clinical studies using transgenic animals, cholinergic agonists, vagotomy, and vagus nerve stimulation have demonstrated this pathway’s role and therapeutic potential in numerous inflammatory diseases. In this review, we summarize what is understood about the inflammatory reflex. We also demonstrate how pre-clinical findings are being translated into promising clinical trials, and we draw particular attention to innovative bioelectronic methods of harnessing the cholinergic anti-inflammatory pathway for clinical use.

## Graphical abstract

**Figure:**
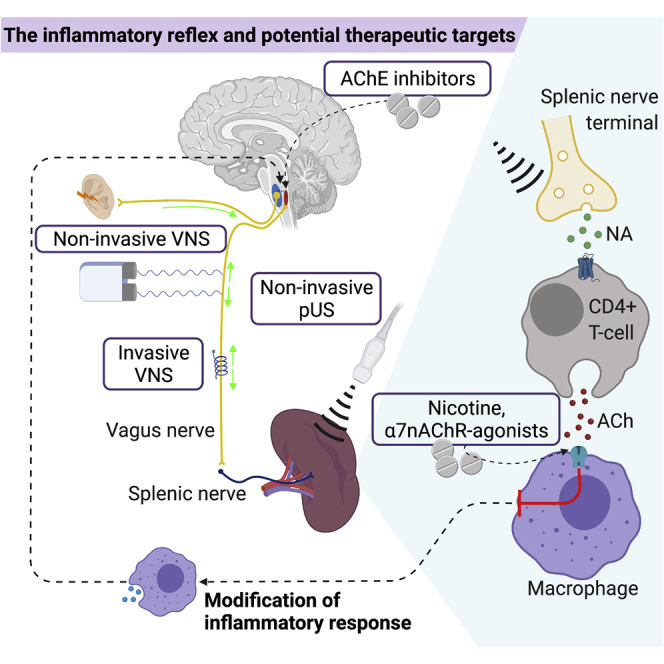

## Teaser

Increasing evidence has revealed the significant anti-inflammatory activity of the cholinergic pathway. Kelly and colleagues review the current knowledge of the neural inflammatory reflex and highlight recent work harnessing the cholinergic anti-inflammatory pathway as a therapeutic strategy targeting disease.

## Introduction

By the end of the 20^th^ century, the key influence of the central nervous system (CNS) on modulating our systemic inflammatory response was well recognized. Cytokines released by immune cells in response to pathogens have the capacity to transmit signals across the blood-brain barrier (BBB) through a variety of mechanisms, including stimulation of the afferent (sensory) vagus nerve.^1^^,^^2^ This stimulates a reciprocal response via the hypothalamic-pituitary-adrenal (HPA) axis.^1^

Building on these initial observations, researchers found that the CNS transmits efferent signals more directly via neural circuits, specifically the efferent vagus nerve of the parasympathetic nervous system, to exert a systemic anti-inflammatory effect.^3^ They termed this the cholinergic anti-inflammatory pathway (CAP) after the acetylcholine-mediated effects of the vagus nerve. The combination of the afferent and efferent arms of this vagal-immune interaction is termed the “inflammatory reflex.”^4^ These seminal observations led to the proposed concept of harnessing the systemic anti-inflammatory activity of the efferent arm of the vagus nerve as a therapeutic platform targeting chronic inflammatory diseases.^5^^,^^6^

The aim of this review is to highlight the historical evidence that supports the concept of harnessing the potential of the parasympathetic nervous system as a complementary anti-inflammatory therapy. In addition, we will describe more recent work translating these observations into the clinical trials arena. In particular, we will highlight the exciting advances in the realm of bioelectronics as potential non-pharmacological therapies.

## The inflammatory reflex

Kevin Tracey and colleagues made the seminal observation that acetylcholine (ACh) and nicotine attenuated pro-inflammatory actions of macrophages. ACh is the key parasympathetic system neurotransmitter. This led to the hypothesis that these ACh-mediated anti-inflammatory effects were mediated via peripheral nicotinic (rather than muscarinic) receptors (nAChR).^3^ The cholinergic vagus nerve is the mediator of the parasympathetic nervous system. *In vivo,* transection of this nerve (vagotomy) in rats subjected to LPS-induced endotoxemia led to a more aggressive systemic inflammatory response, characterized by earlier onset of shock and higher serum and liver levels of the pro-inflammatory cytokine tumor necrosis factor alpha (TNF-α), typically released from macrophages.^3^ Electrical stimulation of the distal arm of the transected vagus attenuated this response.^3^^,^^7^ These findings revealed that the vagus nerve, previously thought only to be activated in response to peripheral inflammation,^1^ was also capable of modulating the inflammatory response through its efferent projections, the now-called cholinergic anti-inflammatory pathway (CAP).^4^

**Figure 1:**
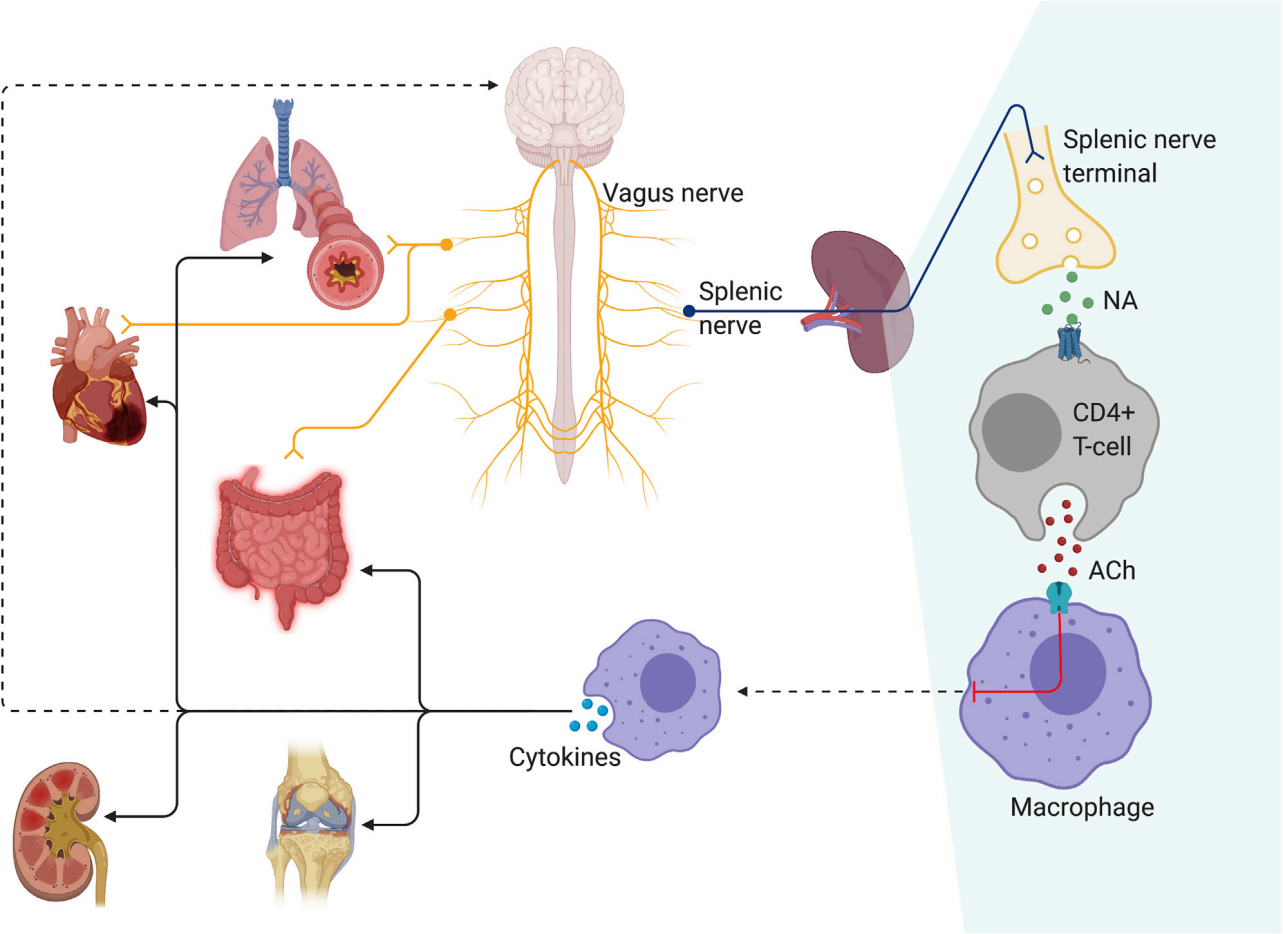
The cholinergic anti-inflammatory pathway Through the inhibition of splenic macrophages, the vagus nerve attenuates inflammatory responses in multiple bodily systems, including the lungs, GIT, myocardium, synovia, and kidneys. The vagus nerve may also mediate some of its effects directly through innervation of viscera (e.g., lungs, heart, GIT). Suppression of the systemic inflammatory response can likewise influence neuroinflammation. ACH, acetylcholine; NA, noradrenaline.

While these initial observations were important, they did not explain the full story. Further animal studies found that splenectomy and transection of the splenic nerve abolished the effects of vagus nerve stimulation (VNS) on systemic TNF-α released in response to endotoxemia and polymicrobial sepsis^8^, ^9^, ^10^ In other words, the vagus nerve modulated the TNF-α response of nAChR-positive splenic macrophages through signals transmitted via the splenic nerve. Specifically, the α7nAChR subtype was responsible for the anti-inflammatory effects of ACh, as demonstrated in α7nAChR knockout (KO) mice.^8^^,^^9^^,^^11^ The splenic nerve is believed to synapse not with macrophages directly (splenic neurons are catecholaminergic, not cholinergic^12^), but instead with choline acetyltransferase-positive (ChAT+), β2-adrenergic-receptor-positive (β2AR+) T cells, which release non-neuronal ACh in response to noradrenaline signaling. Nude mice, devoid of functional lymphocytes, are insensitive to the anti-inflammatory effects of VNS. ChAT+ T cells have been identified at synapses with splenic nerve terminals and are necessary for VNS inhibition of endotoxin-induced TNF-α release.^13^ Furthermore, a series of experiments by Vida and colleagues confirmed β2AR-expressing lymphocytes to be crucial for VNS-induced anti-inflammatory activity.^14^ Nude mice and β2AR KO mice were insensitive to VNS, but the effect was restored by the transferring of β2AR+ T cells into these animal models. The transfer of β2AR KO lymphocytes into nude mice did not restore the effect of VNS. The findings of Rosas-Ballina^13^ and Vida^14^ in combination identify β2AR+, ChAT+ lymphocytes as an essential mediating step between the splenic nerve and macrophages, completing Tracey’s model of the CAP as it is understood today (see Figure 1).^15^

It should be noted that alternative theories to the CAP have been proposed. The concept of a di-synaptic connection between the vagus and splenic nerve has been questioned.^16^ This was based on the observation that VNS did not induce detectable action potentials in the splenic nerves of rats, and an anatomical connection could not be identified at a synaptic level.^17^ This led to an alternative concept that the efferent arm of the inflammatory reflex is not the CAP, but rather the sympathetic nervous system.^17^ Subsequent research demonstrated that action potentials were transmitted via the sympathetic chain and splanchnic nerves, in turn innervating the splenic nerve and ultimately inhibiting cytokine release.^16^, ^17^, ^18^ Another group found that the anti-inflammatory effects of stress and activation of autonomic C1 neurons in the brainstem were not attenuated by subdiaphragmatic vagotomy.^19^ It was similarly found that vagotomy did not exacerbate the effects of endotoxemia, whereas splanchnic neurotomy did.^18^ However, a substantive body of work including Borovikova and colleagues^3^^,^^20^, ^21^, ^22^ has supported the importance of the CAP mediated via the splenic nerve and β2AR-expressing lymphocytes. Martelli and colleagues attributed this discrepancy to an interruption of the HPA axis. However, this failed to explain why stimulation of the distal vagus nerve (which was transected proximally and therefore incapable of transmitting an afferent signal to the hypothalamus) would result in suppression of splenic TNF release without any alteration of corticosterone levels.^3^ Furthermore, the corticosterone antagonist mifepristone did not block the effects of the CAP.^19^ Abe and colleagues showed that VNS could activate the CAP regardless of whether it was applied to the distal or proximal limb of the vagus,^23^ while the contralateral vagus nerve was blocked with lignocaine, potentially suggesting the existence of a second efferent arm to the CAP that could be stimulated via the afferent vagus nerve.

Vida and colleagues^9^ reported that suppression of serum TNF levels in a mouse model of systemic sepsis could also be achieved by splenic nerve stimulation (SNS), and while VNS was dependent on the α7nAChR, SNS was effective in α7nAChR KO mice. Their findings suggested that, although the α7nAChR is an essential component of the CAP at the level of vagus-to-splenic nerve signaling, splenic nerve-to-macrophage signaling may be possible through alternate, α7nAChR-independent mechanisms, though such a mechanism has yet to be identified. Further supporting these findings is the recent finding that while T cells certainly appear capable of forming synapse-like structures with splenic neurons,^24^ and ChAT+ lymphocytes in the spleen are primarily concentrated in the white pulp where sympathetic are situated,^13^ a synaptic connection with ChAT+ lymphocytes could not be identified on confocal microscopy.^25^

These findings suggest that the CAP model may be one of the additional pathways by which our nervous system modulated the systemic inflammatory response. For example, Murray and colleagues have proposed that splenic neurons may communicate with ChAT+ lymphocytes via neurotransmitter diffusion or chemotaxis through the CXCL13 chemokine,^25^ which is upregulated by sympathetic activity, rather than through synaptic transmission. Detailed characterization of alternative or additional pathways within the inflammatory reflex will be essential to developing the therapeutic potential of this field, particularly if these pathways present additional therapeutic targets or help to explain treatment failure in a sub-group of patients. Nevertheless, it is our opinion that there is a convincing and growing wealth of evidence for targeting the vagus nerve and the α7nACh receptor as anti-inflammatory therapies.

As discussed above, the α7nAChR has been identified as the extracellular target of the CAP through KO studies.^8^^,^^9^^,^^11^ The intracellular effects of α7nAChR activation, however, are numerous and not mutually exclusive. In non-neuronal cells, intracellular-signaling cascades are activated via ligand binding of intracellular molecules and tyrosine kinase-mediated increases in intracellular calcium, rather than by ion-channel opening, as seen in neurons.^26^ In macrophages and monocytes, anti-inflammatory effects of VNS are mediated by the recruitment of the tyrosine kinase JAK2 to the α7nAChR and subsequent phosphorylation of STAT3, which blocks cytokine transcription by NF-κB.^27^

**Figure 2:**
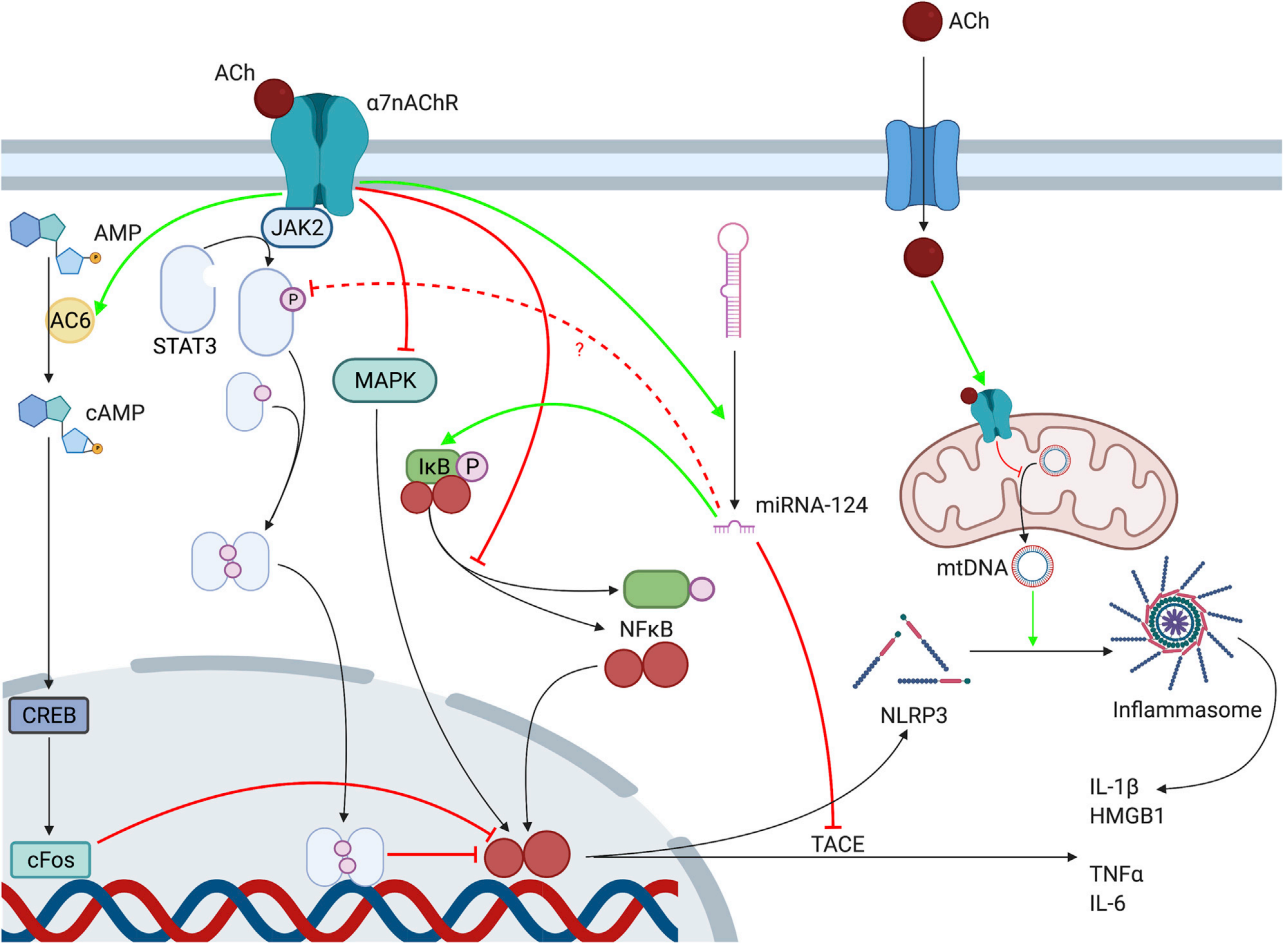
Proposed intracellular mechanisms of the α7nAChR AC6, adenylyl cyclase 6; Ach, acetylcholine; AMP, adenosine monophosphate; cAMP, cyclic adenosine monophosphate; CREB, cAMP response element-binding protein; JAK2, Janus kinase 2; MAPK, mitogen-activated protein kinase; miRNA, microRNA; NF-κB, nuclear factor κ-B; NLRP3, NOD-, LRR-, and pyrin domain-containing protein; STAT3, signal transducer and activator of transcription 3.

The JAK2/STAT3 pathway is not the only one implicated in intracellular signaling of the α7nAChR (see Figure 2). Other molecules have been implicated and may interact with STAT3 or act independently (see Figure 2). These include inhibition of mitogen-activated protein kinase (MAPK) pathways such as ERK1/2,^28^ the activation of adenylyl cyclase (AC) 6, which in turn activates the cAMP-CREB-cFOS pathway,^29^ signaling via heme-oxygenase 1^30^^,^^31^ and heat shock protein (HSP)-70,^32^ and the suppressed phosphorylation of IκB.^33^ Through other pathways, α7nAChR may downregulate cell surface expression of NF-κB-inducing receptors CD14 and Toll-like receptor (TLR)-4^34^^,^^35^ and enhance autophagic activity,^36^, ^37^, ^38^ further contributing to the anti-inflammatory phenotype. ACh, which enters the cytoplasm during states of inflammation, can also act on mitochondrial α7nAChR, preventing the release of mtDNA and the activation of the NLRP3 inflammasome complex responsible for release of cytokines IL-1β and HMGB1.^39^ α7nAChR activation promotes the expression of microRNA-124, which inhibits IL-6 and TNF-α release.^40^ The proposed mechanisms of action of microRNA-124 include targeting of IκB and inhibition of TNF-α-converting enzyme (TACE), but also the *suppression* of STAT3, which was paradoxically found to be an essential mediator of IL-6 production.^40^ Conflicting results of this type demonstrate that elements of this signaling cascade are still poorly understood and that there may be multiple intracellular pathways through which the α7nAChR can act. While the majority of “CAP-targeted” therapies to date are directed toward extracellular components such as the vagus nerve and α7nAChR, further investigation of downstream pathways will help to clarify the mechanism underlying the CAP and may present new therapeutic targets.

## The inflammatory reflex in disease

**Figure 3:**
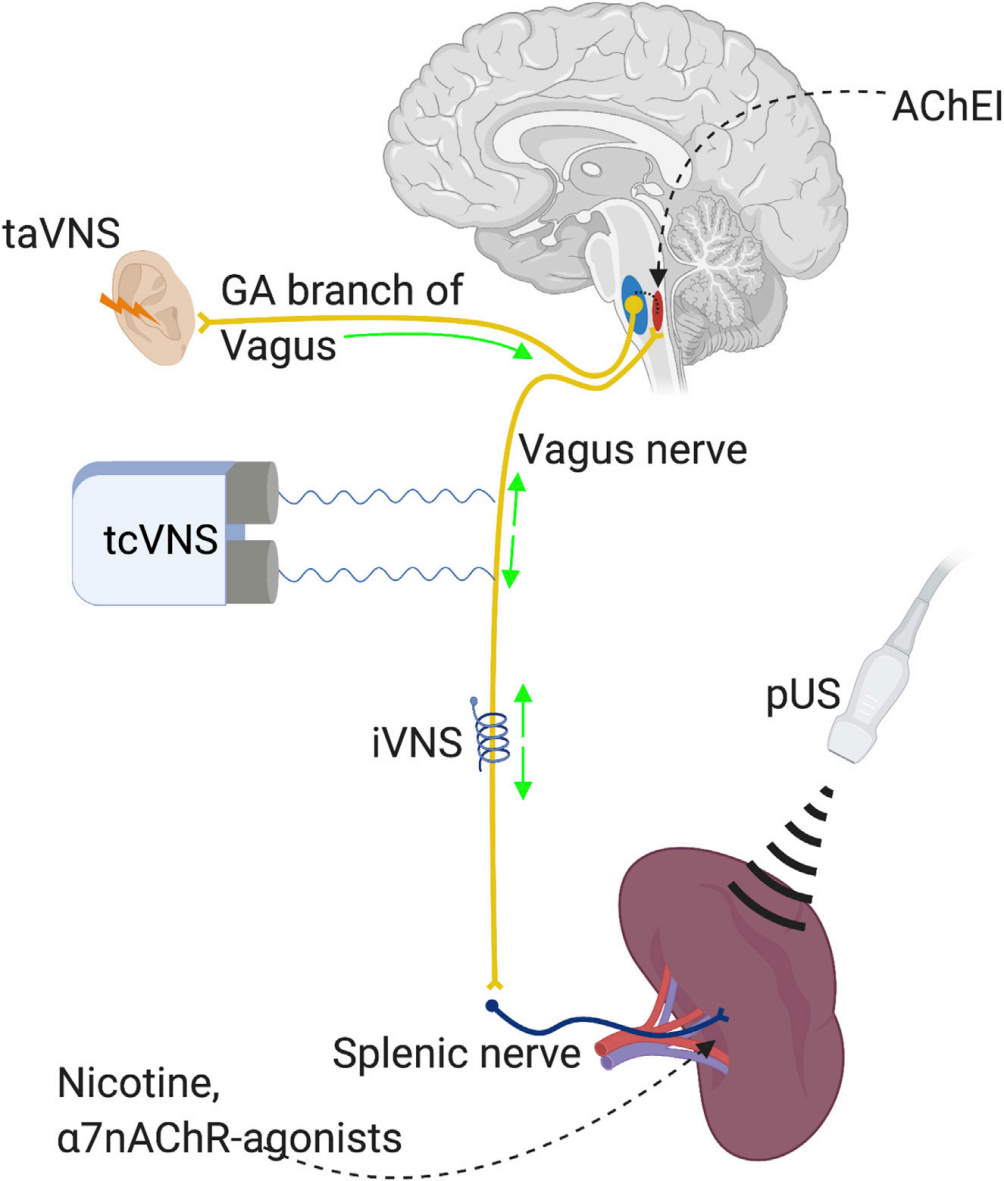
Stimulation of the CAP The CAP can be stimulated pharmacologically through centrally acting acetylcholinesterase inhibitors (AChEI) and peripherally acting nicotine or α7nAChR agnoists. Non-pharmacological stimulation is achieved through invasive and non-invasive VNS or pUS. iVNS, invasive vagus nerve stimulation; taVNS, transauricular vagus nerve stimulation; tcVNS, transcervical Vagus nerve stimulation.

Inflammation is implicated in the pathogenesis of a broad range of human diseases. The demonstration of the significant systemic anti-inflammatory properties of the inflammatory neural reflex makes the CAP an attractive therapeutic target. This could potentially be harnessed pharmaceutically by targeting the α7nAChR or bioelectronically via both VNS and other methods such as splenic ultrasound (see Figure 3). Here, we review evidence on the role of the CAP and its therapeutic potential in inflammatory diseases.

### Rheumatological disease

Rheumatoid arthritis (RA), a chronic inflammatory condition of synovial membranes, and related connective tissue disorders are the most studied with regard to the potential of exploiting the inflammatory reflex therapeutically. Extensive pre-clinical research over the past twenty years has led to more recent exploratory early clinical trials, resulting in an expanding body of work supporting this therapeutic strategy in connective tissue disorders.

Administration of nicotine,^41^ selective α7nAChR-agonist AR-R17779,^42^ and partial agonist GTS-21^43^^,^^44^ mitigate joint swelling^42^, ^43^, ^44^ and reduce both radiological^42^^,^^44^ and histological^41^ measures of bony erosion in animal models of collagen-induced arthritis (CIA). α7nAChR-selective antagonist methyllycaconitine (MLA) attenuated the effect of GTS-21.^43^ These clinical and radiological findings are associated with a significant fall in systemic pro-inflammatory cytokines findings (e.g., TNF-α,^41^, ^42^, ^43^, ^44^ IL-6,^41^, ^42^, ^43^ and IL-1β^44^). Analysis of synovial fluid reveals a parallel fall in pro-inflammatory cytokines with a consequent reduction in joint inflammatory cells^42^ and osteoclasts.^44^

The synovia of CIA mice treated with GTS-21 has significantly reduced expression of CD11c, a relatively specific marker of dendritic cells (DCs), known to be pathogenic in RA.^43^ One potential mechanism for this is that GTS-21 inhibited the differentiation of bone marrow-derived DCs from progenitor cells *in vitro*, and this differentiation was inhibited by MLA.^43^

Nicotine treatment results in lower levels of synovial Th17 cells, a subset of CD4^+^ lymphocytes believed to promote inflammation in RA through secretion of IL-17a.^45^ α7nAChR KO exacerbates CIA with greater levels of joint destruction, higher serum levels of TNF-α and chemokine MCP-1, and a shift to a higher ratio of inflammatory cytokine-producing Th1 cells to IL-10-producing Th2 cells.^46^

Overall, these studies demonstrate that the α7nAChR has anti-inflammatory and potentially disease-modifying effects on RA. A range of cells including macrophages, fibroblasts, T cells, and B cells expressing these receptors can be found in the synovia of patients with RA.^47^ Though it remains to be determined whether infiltration of these cells is actually stimulated directly by ACh or by α7nAChR-independent mechanisms downstream of macrophage activation and cytokine release, or whether α7nAChR expression is purely a secondary marker of inflammatory cell activation.

The vagus nerve has been implicated in RA. Vagotomy was associated with greater levels of synovial neutrophil infiltration and hyperalgesia in a mouse model of antigen-induced arthritis.^21^ Two studies demonstrated that the disease-exacerbating effects of vagotomy were attenuated by α7nAChR stimulation.^42^^,^^48^ While these effects only trended toward statistical significance, the studies used unilateral vagotomy solely, which may, because of compensation from the contralateral nerve, have more modest effects than that of bilateral vagotomy seen in other disease models.^20^ Furthermore, VNS has demonstrated efficacy in the treatment of CIA in at least two studies, one using electrical stimulation^49^ and the other mechanical.^50^ Together, these studies reveal that VNS inhibits joint inflammation and destruction^49^^,^^50^ and release of inflammatory cytokines.^50^ These studies led to a clinical trial of VNS in RA patients.^51^ Eighteen RA patients were implanted with VNS and experienced a rapid improvement in disease severity as measured by the internationally validated disease activity score-28 (DAS28). This DAS is derived from the assessment of 28 specific joints by a healthcare professional combined with both a patient self-reported score for health and well-being and input of blood systemic inflammatory markers (e.g., ESR or CRP). In addition, a corresponding reduction in TNF-α release on lipopolysaccharide (LPS)-whole blood culture was observed, a measure of the systemic pro-inflammatory state, in which blood samples from participants are exposed to an inflammatory trigger *ex vivo*. When VNS was suspended for two weeks, symptoms relapsed but were once again attenuated on reactivation of the stimulator. Recently a sham-controlled pilot study assessed the safety of an implantable VNS device in RA patients but was underpowered to assess clinical efficacy. However, it is worth noting that 5 of 10 patients (seven blinded, three unblinded) in the treatment arm demonstrated a clinically significant change in disease severity score, versus zero out of four sham controls.^52^ Neither of these trials reported serious adverse events, though a high proportion (89% in the former,^51^ 57% in the latter;^52^ 60% of in the treatment arm and 50% of controls) reported mild to moderate side effects. Many adverse events are attributable to procedural complications (i.e., secondary to the surgery itself), but cough and hoarseness are well recognized adverse effects of device activation. In epilepsy, these effects are generally well tolerated by patients and do not limit treatment compliance.^53^ Large-scale placebo-controlled trials are now required to definitively address the therapeutic efficacy of this treatment strategy.

### Gastrointestinal disease

Inflammatory bowel disease (IBD) encompasses ulcerative colitis (UC) and Crohn’s disease (CD), disorders characterized by inflammation of the gastrointestinal tract (GIT). This inflammation is largely mediated by the activation of macrophages and cytokine pathways including TNF-α. The emergence of anti-TNF biologic therapies has transformed the treatment of IBD, particularly CD,^54^ but neither are without risk nor universally effective. Heart-rate variability (HRV) is reduced in IBD,^55^^,^^56^ implying a disruption of vagal tone, which, as discussed in the introduction, can be associated with greater inflammatory activity and represents an attractive target for new therapies.

Evidence for the importance of the inflammatory reflex in IBD comes from *in vivo* studies. α7nAChR KO mice demonstrate more severe responses to dextran sulfate sodium (DSS)-induced colitis, with more severe symptoms, higher disease activity scores, higher tissue and serum levels of cytokines (IL-1β, IL-6, IL-18, and TNF-α), and higher mortality rates,^38^^,^^57^ though not all studies confirm this finding.^58^ α7nAChR-selective agonist PNU282987^59^ and partial agonist encenicline^60^ (the effects of which are significantly attenuated by MLA) inhibit the development of DSS-induced colitis, reflected by less histological damage,^59^^,^^60^ less macrophage infiltration,^59^^,^^60^ and lower tissue levels of cytokines.^59^ Of note, other studies have found α7nAChR activation to be ineffective once DSS-induced colitis is established (i.e., if agonists are administered three days after DSS).^60^^,^^61^ Similar benefits to α7nAChR agonism have been demonstrated in 2,4,6-trinitrobenzene sulfonic acid (TNBS)-induced colitis, a model considered more representative of CD than UC.^60^^,^^62^ However, an earlier study found two agonists (AR-R17779 and GSK1345038A) to be ineffective in TNBS- and DSS-colitis; low doses were paradoxically harmful whereas high doses were protective.^63^ The authors theorized that higher agonist doses might have α7nAChR-independent effects. This hypothesis requires replication and further investigation.

Vagotomy was found to be associated with a 50% greater risk of IBD in a recent epidemiological study including over 15,000 vagotomized patients and more than 600,000 age-matched controls.^64^ A series of comprehensive experiments by one research group further demonstrated this phenomenon. Galantamine, an acetylcholinesterase inhibitor (AChEI), has demonstrated promising results in preventing the induction of TNBS-^65^ and DSS-induced colitis,^66^ an effect mediated by central muscarinic (mAChR) activation of the CAP^65^ and dependent on the vagus nerve and α7nAChR. Vagotomy leads to an increase in colitis severity and tissue cytokine levels in mouse models of DSS- and dinitrobenzene sulfonic acid (DNBS)-induced colitis.^22^ Similar effects were seen following splenic denervation and splenectomy.^66^ However, VNS, which has demonstrated efficacy in models of IBD,^67^ has also been shown to reduce bowel inflammation independently of splenic innervation, and instead via cholinergic stimulation of α7nAChR-expressing resident macrophages in the gut.^68^ This pathway has recently been termed the “enteric-CAP.”^69^ Thus, the vagus nerve once again appears key to the α7nAChR-mediated effects in IBD. Further research has suggested that the vagus nerve is capable of inducing α7nAChR-independent anti-colitic effects, such as by the recruitment of regulatory T cells (Tregs).^58^

To date, there have been few published clinical trials of cholinergic agonists in IBD. Studies have shown that transdermal nicotine provides clinically meaningful benefits as an adjuvant therapy in active UC.^1^ These trials were based on the hypothesis that nicotine is the causative agent behind the inverse relationship between smoking and UC risk and predate discovery of the CAP. The effect of nicotine on UC remains unexplained, and while it is tempting to imagine a mechanistic role for the α7nAChR between nicotine and UC, such a hypothesis would require further and more specific investigation. It is also important to note that smoking has the opposite relationship with CD, and some have even proposed that nicotine may be responsible for this effect via immunomodulatory mechanisms, though this lacks convincing evidence.^70^ Semapimod (a.k.a. CNI-1493), a small molecule that centrally activates the CAP,^7^ failed to reduce severity in CD patients over placebo after three doses. It did demonstrate intra-patient improvements after repeated dosing in an open-label continuation trial, but a high rate of infusion site reactions limited its tolerability.^71^ Large-scale trials are required to more accurately define responders while limiting systemic side effects in this patient population.

Because such off-site effects may prevent the delivery of α7nAChR agonists at the doses required for clinical benefit, efforts have therefore been made to target the CAP more directly using VNS. Two small open-label trials,^72^ one published in abstract form only,^73^ have trialed VNS in a total of 23 CD patients, reporting clinically meaningful improvements in clinical and endoscopic disease scores. However, a relatively high number (9–11) of cases experienced worsening of disease. Again, more substantive clinical trial data is required to evaluate the efficacy and safety of VNS in IBD.

### Lung disease

In a mouse model of acid-induced acute lung injury,^74^ markers of inflammation, including excess lung water, lung vascular permeability, and bronchoalveolar lavage levels of leukocytes, were significantly reduced by the administration of nicotine, choline, and α7nAChR-selective agonist PNU-282987. These acid-induced effects were enhanced in α7nAChR KO mice. Leukocytes expressed higher cytoplasmic levels of NF-κB, and this effect was abrogated by treatment with nicotine. Vagotomy exacerbates the inflammatory response in animal models of ventilator-induced lung injury (VILI).^20^ Stimulation of the inflammatory reflex using α7nAChR agonists^20^^,^^74^^,^^75^ or VNS^20^^,^^75^ mitigated these inflammatory responses. α7nAChR agonist GTS-21 proved effective in reducing both organ injury and inflammatory markers in radiation-induced lung injury.^76^ Recently, neostigmine, an AChEI, demonstrated efficacy in a model of allergic asthma. This effect was associated with an increased expression of α7nAChR in the lungs.^77^

These studies present a strong case for the role of the CAP in lung inflammation and justify further study of the vagus nerve and its effects in the lungs. However, the relationship between the vagus nerve and the lungs may be more complex than that seen in inflammatory diseases of other organs. Activation of mAChR has bronchoconstrictive and possibly pro-inflammatory effects in the lungs.^78^ The use of beta-adrenergic agonism and muscarinic antagonism in the symptomatic treatment of COPD and asthma is based on this very concept. On the other hand, activation of other α7nAChR+ airway mucosa cells (e.g., type 2 innate lymphoid cells [ILC2s] and pulmonary neuroendocrine cells [PNECs]) downregulates the production of pro-inflammatory cytokines.^79^ It is possible that vagal release of ACh, which is capable of activating not only the α7nAChR but also other ACh receptors, in the airway mucosa could activate both pro- and anti-inflammatory processes. There are therefore two important clinical questions: how do these two pathways behave together *in vivo*, and does the balance tip toward a pro- or anti-inflammatory endpoint when there is an increase in vagal tone?

One study found that VNS inhibited LPS-induced TNF production in cardiac and hepatic tissues, but not in the lungs.^80^ However, other studies^20^^,^^75^ have found VNS to be protective against VILI and to impair anti-inflammatory processes in the lungs including IL-6 release.^20^ So, while evidence is variable, there is an increasing body of work supporting the hypothesis that vagus nerve activity mitigates inflammation in the lungs as it does in other organs, though whether this effect results solely from the spleen-mediated CAP or is also influenced by direct parasympathetic innervation of the airways warrants further investigation.

α7nAChR are expressed by various cells within lung mucosa.^81^ The downstream effects of α7nAChR activation in lung tissue are heterogeneous, complex, and remain to be fully elucidated but are important to consider when targeting the α7nAChR for therapeutic purposes. It has been postulated on the basis of *in vitro* studies and some animal models that α7nAChR activation may increase the metastatic potential of lung cancers^81^ and fibrogenesis in pulmonary fibrosis.^80^^,^^82^ However, these hypotheses lack clinical evidence to date. In fact, vagotomy has associated with a higher risk of lung and other cancers in pre-clinical and human epidemiological studies.^79^ Therefore, the CAP may actually have anti-cancer properties.

While there are some conflicting studies and unanswered questions, the trend of available evidence indicates that the CAP has therapeutic potential in lung disease, warranting further pre-clinical and clinical study. Other inflammatory lung diseases such as sarcoidosis, in which TNF-α-release from macrophages is central to the pathogenesis,^83^ also present tempting targets for future interventions.

### CNS disease

Underlying inflammation forms the basis for the pathogenesis of many CNS diseases. These include not only the classic “inflammatory” disorders such as encephalitis and multiple sclerosis (MS), but also degenerative diseases such as Alzheimer’s disease (AD) and Parkinson’s disease (PD) and psychiatric disorders including depression and schizophrenia. The CNS immune system, which was once considered immune-privileged, operates somewhat independently, but not totally isolated, from the peripheral system. The BBB, under normal physiological conditions, limits the influence of peripheral immune cells on the CNS. A specialized subset of macrophages known as microglia govern innate immunity.^84^ The α7nAChR appears to play an important role in modulating the neuroinflammatory response in CNS diseases; the clinical use of VNS in epilepsy and depression are therapeutic examples of this. However, as we will demonstrate, the intricacies of the CNS immune system add an extra layer of complexity to its relationship with the CAP.

ACh is a ubiquitous and multifunctional neurotransmitter of the CNS, and α7nAChR are expressed abundantly on neuronal and non-neuronal cells, including microglia, astrocytes (CNS glial cells), BBB endothelial cells, and oligodendrocyte precursors (responsible for myelin production).^85^ Microglia mediate inflammatory processes through the release of TNF-α, amongst other mechanisms, and are sensitive to the anti-inflammatory effects of α7nAChR activation,^85^ presenting a suitable target for the CAP.

The risk of AD correlates with genetic variation in the α7nAChR.^86^ α7nAChR KO mice exhibit enhanced depression-type behaviors,^87^ and in a transgenic model of AD, experience fewer deficits and less neurodegeneration.^88^ In an ischemic stroke model, α7nAChR KO confers smaller infarct size with corresponding preservation of neurological function.^89^ One study found that α7nAChR KO was protective against experimental autoimmune encephalomyelitis (EAE),^90^ the gold-standard rodent model of MS. Two other studies found that KO did not alter the EAE phenotype.^91^^,^^92^ Though all three found that nicotine was protective against EAE, α7nAChR KO attenuated this protection.^90^, ^91^, ^92^ The reason proposed for this discrepancy was that α7nAChR activation not only mediates the migration and activation of pathogenic Th1 and Th17 cells in EAE, but also the actions of antigen-presenting cells (APCs) within the CNS, which are necessary to trigger EAE.^90^ Other research suggests that nicotine confers some of its anti-inflammatory effects through other nicotinic-receptor subtypes,^91^ including α9nAChR.^90^^,^^93^ Nevertheless, PNU-282987 has demonstrated clinical efficacy in EAE, improving clinical severity scores, reducing leukocyte infiltration into the CNS, and reducing mRNA expression of IL-6, IL-1β, IL-18, and TNF-α while also inducing autophagy by microglia and splenic macrophages. Thus, while α7nAChR may have a number of heterogeneous and possibly opposing functions, therapeutic strategies targeting the receptor are likely to be neuroprotective in EAE based on current evidence.

Pharmacological α7nAChR activation has also demonstrated therapeutic potential in models of other CNS disorders including AD,^94^ PD,^95^ schizophrenia,^96^ ischemic stroke,^97^ intracerebral haemorrhage,^98^ LPS-induced anxiety and depression,^99^ traumatic brain injury (TBI),^100^ and cardiopulmonary bypass-induced brain injury.^101^ AChEIs are licensed for use in improving cognition in AD. Historically, clinical efficacy of AChEIs was thought to be mediated via increasing synaptic concentrations of ACh and thus compensating for the loss of cholinergic neurons. However, it now appears likely that at least some of the clinical benefit is conferred through its anti-inflammatory effect.^102^ That being said, at least three α7nAChR-selective agonists have been studied in clinical trials of human AD patients, but none have progressed past stage two, either because of adverse effects, insufficient clinical benefit, or without explanation.^103^

The only diseases in which VNS is currently licensed for clinical use are epilepsy, depression, and headache, fundamentally CNS disorders. There is emerging clinical evidence for efficacy in other CNS disorders including AD^53^ and stroke.^104^ VNS has proven to be very efficacious in reducing seizure frequency in treatment-refractory epilepsy,^105^ though its mechanism of action is not understood. It was first used in epilepsy over 30 years ago, long predating characterization of the CAP, and was thought to directly inhibit the electrical activity of partial seizures. Soon thereafter, research showed that chronic intermittent stimulation induced long-term neural network changes, reducing seizure frequency.^106^

VNS demonstrates disease-modifying effects in pre-clinical models of schizophrenia by restoring normal neuronal activity and by reversing the hypersensitive amphetamine psychomotor response.^107^ It has been shown to enhance the rate of recovery after established ischaemic^108^ and haemorrhagic^109^ stroke. These effects might be seen as neuroplastic rather than neuroprotective. Cholinergic circuits and microglia are believed to have important neuroplastic properties.^110^ It is not clear from these studies that VNS exerts its effect through anti-inflammatory mechanisms or via the α7nAChR. One might expect that CNS inflammation would activate intrinsic cholinergic circuits and directly modulate inflammation without the unnecessary steps of involving the vagus nerve and spleen. Certainly, microglia appear susceptible to intrinsic cholinergic circuits with anti-inflammatory and neuroprotective effects.^109^ However, there is evidence to support a role for VNS in neuroinflammation and that CAP suppression of systemic inflammation could mediate this.

VNS reduces CNS levels of inflammatory cytokines and other biomarkers in models of systemic endotoxaemia.^110^^,^^111^ In the context of addressing was that this observed VNS anti-inflammatory activity a peripheral response, investigators have shown that this anti-inflammatory CNS effect is inhibited in vagotomized mice, supporting the role of the efferent vagus mediating these effects on neuroinflammation.^112^ Supporting this hypothesis, splenectomy impaired the anti-inflammatory effects of α7nAChR-agonism in TBI.^100^ Again, this may not be the most suitable model for organ-specific neuroinflammation, as TBI is accompanied by a systemic inflammatory response and translocation of peripheral immune cells across the disrupted BBB.^100^ Systemic inflammation is associated with deleterious effects in most neurodegenerative disorders.^112^ So, it is plausible that VNS would have neuroprotective effects mediated via the efferent nerve.

## Other systems

The cholinergic system, which is found in both neuronal and non-neuronal cells, mediates complex functions in all organs of the body. Therefore, it is plausible that manipulation of the CAP would have therapeutic potential in chronic inflammatory end-organ injury. In a comprehensive set of experiments, Inoue and colleagues^23^ illustrated the capabilities of the CAP in protecting the kidney from renal ischemia-reperfusion injury (IRI). VNS, when applied 24 or 48 h before ischemia, attenuated cytokine response and acute kidney injury. This effect was not seen in α7nAChR KO or splenectomized mice. The efferent vagus nerve does not innervate the kidney,^113^ and blocking of the sympathetic renal nerve actually prevented renal IRI,^23^ so VNS appears to exert its reno-protective effect indirectly through the spleen.

Deletion of the α7nAChR results in larger infarct size and greater inflammatory response in mice subjected to myocardial infarction.^36^ α7nAChR agonists^114^ and VNS^115^ have the opposite effect. A non-invasive form of transcutaneous VNS, discussed below and in Table 1, has demonstrated cardioprotective effects during acute myocardial infarction in a human randomized control trial (RCT).^116^ Unlike the kidney, the heart receives extensive parasympathetic input from the vagus nerve, though whether the cardioprotective effects of VNS are mediated via direct myocardial innervation, the splenic CAP, or both has not been investigated.

**Table 1:** Clinical trials using electrical stimulation of the CAP

Treatment	Population	Reference	Findings
iVNS	rheumatoid arthritis	Koopman et al., 2016^52^	n = 18 RA patients were implanted with VNS and stimulated up to four times daily. Stimulation was associated with a reduction in disease activity (DAS28) and impaired TNF release on LPS-whole blood culture. These measures relapsed when stimulation was suspended for 14 days but improved again after reactivation. Clinical improvement was maintained at 84 days post-implantation.
	Crohn’s disease	Sinniger et al.,^74^ 2020	n = 9 patients with active CD receiving azathioprine or no treatment were implanted with VNS. Over twelve months, five experienced improvement in symptomatic (CDAI) and six in endoscopic (CDEIS) measures of severity. Two experienced worsening of disease and were removed from the study.
	rheumatoid arthritis	Genovese et al.*,* 2020^53^	n = 14 treatment-refractory RA patients. 3 received treatment in an open label pilot study. The remaining 10 were randomized to receive 1 min of stimulation daily (n = 3), four times daily (n = 4), or sham procedure (n = 4) using a novel design of VNS device. 5 of 10 actively treated subjects demonstrated clinical improvement versus no controls. There was a significant reduction in cytokine (IL-1β, IL-6, and TNF) response to LPS-whole blood culture in the treatment group. MRI features of RA did not improve. One case of transient Horner’s syndrome and another of transient vocal cord paralysis, amongst other adverse effects, were reported.
TcVNS	healthy participants	Lerman et al., 2016^117^	n = 20 (10 tcVNS and 10 sham controls, randomized). 3 courses of tcVNS over one day (2 min to each Vagus nerve per course) significantly reduced cytokine (TNF-α, IL-1β) and chemokine (MIP-1α, MCP-1, IL-8) response to LPS-whole blood culture compared to baseline and to controls.
	healthy participants	Brock et al., 2016^118^	n = 20 (internal controls). A single course of 120 s of tcVNS to each vagus nerve was sufficient to induce a small but significant reduction in circulating TNF-α levels, but no other cytokines, after 24 h. Blood samples were not challenged with LPS.
	Sjögren’s syndrome	Tarn et al., 2018^119^	n = 15 female participants (internal controls). 3 weeks of twice daily tcVNS was associated with (1) improvement in fatigue score (n = 12/15), (2) reduced cytokines (TNF-α, IL-6, IL-1β, IP-10) and chemokine (MIP1α) response to LPS-whole blood culture, and (3) a transient rise in circulating T cells, NK cells, and NKT cells after first administration only.
	rheumatoid arthritis	Drewes et al.*,* 2020^120^	n = 36, 16 with active RA and 20 with low activity RA. 120 s of tVNS three times daily for four days was associated with a significant reduction in DAS28-CRP and IFN-γ in non-stimulated blood (but not other cytokines) in the active group only. There was also a statistically significant reduction in blood pressure in this group, possibly indicative of vagus nerve activity. Surprisingly, the low activity group actually experienced a reduction in cardiac vagal tone and in serum levels of IL-10.
TaVNS	impaired glucose tolerance	Huang et al., 2014^121^	n = 35 who received 12 weeks of taVNS experienced a reduction in fasting plasma glucose, 2-h plasma glucose, and Hba1c compared with n = 30 receiving no treatment in a parallel non-randomized observational study. However, a sham-placebo group (n = 35) experienced a similar reduction in 2-h plasma glucose and Hba1c.
	acute STEMI and MIRI post-PCI	Yu et al., 2016^122^	n = 95 (47 taVNS, 48 sham-controls, randomized). TaVNS was applied before and throughout percutaneous coronary intervention (PCI). Intervention group demonstrated (1) fewer arrhythmias, (2) more favorable echocardiographic features, and (3) lower levels of serum cardiac enzymes and cytokines (TNF-α, IL-6, IL-1β, HMGB-1).
	rheumatoid arthritis and healthy participants	Addorisio et al., 2019^123^	Two days of twice-daily taVNS was associated with lower cytokine levels on LPS-whole blood assay in two separate studies (n = 9, TNF-α assay. n = 19 TNF, IL-1β, and IL-6 assays). n = 9 patients with RA experienced a significant reduction in disease activity (DAS28), sustained for at least one week after treatment.
	systemic lupus erythematosus	Aranow et al., 2021^124^	n = 18 (12 taVNS and 6 controls) received 5 min of taVNS or sham procedure daily for four days in a double-blinded RCT. One subject was excluded and replaced due to a respiratory tract infection. 83.3% of taVNS participants experienced a meaningful reduction in subjective measurements of pain and fatigue at 12 days versus 16.7 and 0% of controls, respectively. However, improvements in objective measures by blinded physicians of disease activity were not statistically significant, nor were inflammatory markers or cytokine levels.

iVNS, invasive VNS; taVNS, transauricular VNS; tcVNS, transcervical VNS; SLE, systemic lupus erythematosus.

Vagotomy has an exacerbating effect on models of pancreatitis.^125^ Stimulation of the CAP with α7nAChR agonists^125^ or centrally acting agents^122^ attenuates the disease process.

## Clinical applications and future directions

As the therapeutic potential of the CAP becomes increasingly realized in pre-clinical studies, efforts have begun to translate this work into new clinical therapies (see Figure 3).

Attempts at pharmacological stimulation of the CAP have had mixed success. As discussed above, an RCT of Semapimod in CD failed to meet its primary endpoint, and the medication was poorly tolerated.^73^ A pilot study of GTS-21 in 14 healthy volunteers failed to attenuate the inflammatory response in LPS-induced endotoxemia *in vivo*. The pharmacokinetics of GTS-21 were found to vary greatly between participants in this study. It is possible that insufficient doses were used and it is likely that the study was underpowered.^126^ On the other hand, an RCT of galantamine successfully reduced serum levels of TNF-α, increased levels of IL-10, and improved insulin resistance in patients with metabolic syndrome.^127^ α7nAChR and other AChR have extensive non-immune actions on other cell types. These actions could be responsible for some of these agents’ adverse effects, limiting their clinical use.

Bioelectronic therapies, which target the CAP more specifically, such as VNS, offer an attractive alternative to drug-based therapies modulating cellular α7nAChR expression. Pilot studies of invasive VNS (iVNS), which have shown promise in RA^51^^,^^52^ and IBD,^72^ are described above and in Table 1. As already discussed, VNS implantation is generally well tolerated but is not without adverse effects. Patients may be reluctant, or even physically unsuitable, to undergo implantation. Implantation may be impractical and unjustified in acute or monophasic illnesses which require urgent, but not long-term, intervention and therefore would be more suited to a temporary form of immunomodulation. In recognition of these limitations, but staying cognizant of the significant burden of chronic inflammatory diseases, the industry has expressed significant interest in developing non-invasive bioelectronic devices for these diseases.

The vagus nerve can be stimulated transcutaneously (tVNS) by placing an electrode over the cervical vagus nerve in the neck (transcervical VNS, tcVNS) or the auricular branch in the cymba concha of the external ear (transauricular VNS, taVNS). A device for the former is FDA approved for use in migraine, though efficacy was only demonstrated in a sub-group analysis of patients adherent to treatment,^128^ so patient concordance may limit the efficacy of such a treatment in practice. More recently, the FDA fast-tracked approval for tVNS use in treating respiratory symptoms of COVID-19.^129^ Stimulation of the afferent vagus nerve with taVNS activates vagal brainstem nuclei (see Figure 3).^130^^,^^131^ It is proposed that signals in these nuclei are, in turn, relayed to the efferent vagus nerve based on the observation that taVNS can have systemic autonomic effects. Certainly, both tcVNS and taVNS have demonstrated anti-inflammatory effects in an animal model of endotoxaemia.^132^ These effects were eliminated by both vagotomy and an α7nAChR antagonist, suggesting involvement of the CAP. TaVNS has also demonstrated anti-inflammatory and disease-modifying effects in models of post-operative ileus.^130^ Neuroprotective effects have been demonstrated in PD,^133^ post-operative cognitive dysfunction,^134^ and ischemic stroke.^131^ In more recent studies, such effects were associated with a reduction in intracerebral cytokine release and an upregulation of α7nAChR expression.^133^^,^^134^ Intracerebral upregulation of the α7nAChr has also been observed in response to PNU-282987 with apparent anxiolytic and anti-depressant effects.^135^

Table 1 summarizes the findings of human clinical trials aimed at harnessing the CAP using iVNS and tVNS. Several other tVNS trials not listed in Table 1 have been conducted,^136^ for example in conditions for which iVNS has already been approved (epilepsy and migraine) and in patients with other neurological conditions (e.g., depression and PD) where symptomatic improvement has been demonstrated. However, unlike the conditions listed in Table 1, the anti-inflammatory role of VNS in these neurological disorders remains less well defined and is not typically measured in trial outcomes, so the effects of tVNS may or may not be anti-inflammatory. One short, double-blinded pilot study in systemic lupus erythematosus (SLE) showed improvement in only subjective markers of disease but not in objective measures or inflammatory markers.^137^ However, the overall evidence from these studies, though small in size and number, supports a potential therapeutic benefit for both iVNS and tVNS across a range of inflammatory disorders, warranting larger RCTs.

Recently, an innovative method of stimulating the CAP using non-invasive pulsed ultrasound (pUS) was proposed.^138^ Administration of abdominal ultrasound was found to attenuate subsequent renal IRI in rats. This effect was dependent on functional CD4^+^ T cells^139^ and was not observed following splenectomy, KO or inhibition of the α7nAChR, or splenic denervation.^124^ Prevention of acute kidney injury (AKI) was associated with impaired inflammatory potential of splenic leukocytes and reduced levels of serum and renal cytokines including TNF-α and IL-6.^124^ It is believed that the pUS stimulates the CAP downstream of the vagus nerve in the spleen. Focusing pUS waves at the site of splenic nerve terminals stimulates the release of noradrenaline and ACh in a process dependent on ChAT+ CD4^+^ T cells and the α7nAChR.^138^ Splenic^138^^,^^140^^,^^141^ or abdominal^142^ pUS has proven effective in preventing the effects of endotoxaemia,^138^ prolonging survival in sepsis^141^ and reversing the effects of established inflammatory arthritis^140^ and DSS-colitis.^142^ pUS applied to the spleen or neck reduces infarct size 3-fold in myocardial ischemia-reperfusion injury exacerbated by hyperglycaemia.^143^ Focused pUS may also have anti-inflammatory uses at other sites. The afferent fibers of the vagus nerve be stimulated at the porta hepatis in the liver, modulating hypothalamic insulin sensitivity resulting in attenuation of hyperglycaemia^138^ with associated reduction in weight gain and an overall down-regulation of hepatic inflammation.^69^ Together, these results present a potential therapeutic role in modulating the CAP across a range of organ systems.

In addition to being non-invasive and apparently safe, a theoretical advantage to splenic pUS is that it avoids the non-CAP effects on other organs in which the vagus has been implicated, such as in the lungs.^79^^,^^82^ By the same token, however, limiting the CAP effects to those mediated by splenic macrophages could be less efficacious in conditions such as IBD, where vagal innervation of the GIT might play a role.^68^ The effects of pUS on DSS-colitis, administered non-specifically throughout the abdomen, were almost absent in splenectomized mice, aside from a mild improvement in bloody stool frequency and evidence of AChR+ cell recruitment in the mesenteric lymph node.^142^ Recently, however, pUS was shown to be effective in DSS-colitis by stimulating the CAP at the level of the celiac ganglion.^144^ Human clinical trials of pUS in inflammatory disease are now warranted to assess its therapeutic potential and answer these questions.

## Conclusion

The inflammatory reflex is a pervasive homeostatic mechanism that can influence inflammatory diseases across all bodily systems. The nature of certain interactions between the CAP and individual organs remains unclear, as with the CAP’s downstream cellular mechanisms. Nevertheless, evidence to date presents the CAP as an enticing therapeutic target for a wide range of diseases. Findings of pre-clinical experiments are now being translated into small but promising clinical trials. Pharmacological manipulation of the CAP remains somewhat elusive but warrants further study. Bioelectronic techniques capable of harnessing the CAP through invasive and non-invasive methods such as VNS and splenic pUS have shown promising results and may present an innovative form of anti-inflammatory therapy.
